# Neural Substrates of Poststroke Depression: Current Opinions and Methodology Trends

**DOI:** 10.3389/fnins.2022.812410

**Published:** 2022-04-06

**Authors:** Chensheng Pan, Guo Li, Wenzhe Sun, Jinfeng Miao, Xiuli Qiu, Yan Lan, Yanyan Wang, He Wang, Zhou Zhu, Suiqiang Zhu

**Affiliations:** Department of Neurology, Tongji Hospital, Tongji Medical College, Huazhong University of Science and Technology, Wuhan, China

**Keywords:** poststroke depression, neural substrate, lesion analysis, gray matter atrophy, regional brain activity, brain network, lesion-network mapping, disconnectome

## Abstract

Poststroke depression (PSD), affecting about one-third of stroke survivors, exerts significant impact on patients’ functional outcome and mortality. Great efforts have been made since the 1970s to unravel the neuroanatomical substrate and the brain-behavior mechanism of PSD. Thanks to advances in neuroimaging and computational neuroscience in the past two decades, new techniques for uncovering the neural basis of symptoms or behavioral deficits caused by focal brain damage have been emerging. From the time of lesion analysis to the era of brain networks, our knowledge and understanding of the neural substrates for PSD are increasing. Pooled evidence from traditional lesion analysis, univariate or multivariate lesion-symptom mapping, regional structural and functional analyses, direct or indirect connectome analysis, and neuromodulation clinical trials for PSD, to some extent, echoes the frontal-limbic theory of depression. The neural substrates of PSD may be used for risk stratification and personalized therapeutic target identification in the future. In this review, we provide an update on the recent advances about the neural basis of PSD with the clinical implications and trends of methodology as the main features of interest.

## Introduction

Poststroke depression (PSD) is a common complication of stroke, involving about 29% of patients at any time within 5 years poststroke (Ayerbe et al., 2013). PSD is associated with heavier healthcare burden, poorer functional outcome, and higher long-term mortality in stroke survivors (Robinson et al., 1986; Ghose et al., 2005; Jia et al., 2006; Ayerbe et al., 2013; Bartoli et al., 2013; Kutlubaev and Hackett, 2014). To aid in early prediction and effective intervention for PSD, its etiology and mechanisms need to be scrutinized. Based on accumulating evidence, it is generally believed that PSD has underlying neurobiological causes and is not only a psychosocial response to the new disability or stressful life event (Folstein et al., 1977; Towfighi et al., 2017; Mayman et al., 2021). The association between lesion location and PSD first reported in the 1970s (Robinson et al., 1975, 1983; Robinson and Price, 1982), although extensively investigated as a potential biological factor of PSD, is still of considerable debate (Nickel and Thomalla, 2017; Towfighi et al., 2017). Most of those studies were of traditional design with relatively low spatial accuracy for visual lesion localization (Nickel and Thomalla, 2017). Until 2003, a new technique called voxel-based lesion-symptom mapping (VLSM) was developed to test the lesion–behavior correlation at the voxel level and has been widely used to identify the neural substrates of symptoms after focal brain damage such as PSD, poststroke cognitive impairment, poststroke dysphagia, and stroke-related myocardial injury (Bates et al., 2003; Ay et al., 2006; Kim et al., 2017; Hess et al., 2021; Weaver et al., 2021a). Moreover, there is a growing consensus that the rough lesion location used in traditional lesion analysis and the spatial topography information used in VLSM only represent the “tip of the iceberg”: a surface-level depiction of the lesion largely blind to its impact on the underlying extensive brain networks (Catani et al., 2012; Fox, 2018). The brain network dysfunction caused by a given stroke lesion, instead of the lesion itself, might be the key neural substrate of poststroke symptoms especially when patients with the same symptom have lesions in various brain regions with little lesion overlap (Fox, 2018). Thanks to advances in neuroimaging techniques and statistical methodologies in the past two decades, lesion-symptom inference in high spatial resolution, with robust statistics and from the perspective of the human connectome, can be performed and new evidence for the neural substrates of PSD, beyond those findings from traditional studies, is emerging. In this review, we provide a brief summary of traditional studies and an update on the latest advances about the neural basis of PSD, which may shed light on the etiology, prediction, therapeutic target identification, and future research directions for PSD.

## Definition, Diagnosis, and Measurement of Poststroke Depression

The Diagnostic Statistical Manual of Mental Disorders-5 (DSM-5), as gold standard for PSD diagnosis, defines PSD as “depressive disorder due to another medical condition” with depressive features, major depressive-like episode, or mixed-mood features (American Psychiatric Association, 2013). Some aspects in DSM-5 criteria, such as “disturbance is the direct pathophysiological consequence of another medical condition,” are almost impossible to prove in clinical practice (American Psychiatric Association, 2013). Therefore, the diagnosis of PSD relies on a thorough clinical interview with careful exploration of presenting symptoms and is commonly assisted by the use of screening tools (specifically the depression rating scales validated in PSD screening) (Chun et al., 2021). Although multiple depression scales have been applied and compared in previous studies, the optimal screening tool for PSD remains undetermined (Towfighi et al., 2017). One meta-analysis reported three scales as promising options with relatively high sensitivity and specificity in PSD screening: Center of Epidemiological Studies-Depression Scale (CESD), Hamilton Depression Rating Scale (HDRS), and Patient Health Questionnaire (PHQ)-9, compared with other scales including Geriatric Depression Scale (GDS), Hospital Anxiety and Depression Scale (HADS), and PHQ-2 (Meader et al., 2014). Another study found that HDRS, Beck Depression Inventory (BDI), and Clinical Global Impression (CGI) assessment by professionals showed similar performance in PSD screening (Berg et al., 2009). Of note, the optimal rating scale along with the optimal cutoff value for PSD diagnosis may vary across different stages after stroke (Berg et al., 2009), which warrants further investigation. The methods for PSD diagnosis or measurement in previous studies on the neural substrates of PSD are summarized in Table 1.

**TABLE 1:** Study design and findings for studies on neural substrates of PSD.

Study	Imaging biomarker	Timing of behavioral assessment since lesion onset	Behavioral assessment method	Behavioral outcome	Regions or networks implicated	Results involving PFC
**Traditional ROI analysis**						
Nickel and Thomalla, 2017 *	Lesion location	Varied*	Varied*	Varied*	Varied*	Yes, frontal lobe reported in 7/17 studies
Koenigs et al., 2008	Lesion location	More than 3 months	BDI-II, DSM-4, and NRS for one dataset; BDI-IA or BDI-II for another dataset	Ordinal (low, intermediate, or high severity)	Bilateral DLPFC	Yes
**Lesion subtraction analysis**						
Kim et al., 2017	Lesion location	Within 3 months	DSM-4 and GDS	Binary PSD diagnosis (DSM-4 met and GDS > 16)	Inferior posterior lobe of the left cerebellar hemisphere	PFC not covered in study design
**VLSM**						
Gozzi et al., 2014	Lesion location	Within 12 days and at 1 month poststroke	DSM-4 and HADS	Binary PSD diagnosis (DSM-4 met and HADS > 11)	Negative	No
Kim et al., 2017	Lesion location	Within 3 months	DSM-4 and GDS	Binary PSD diagnosis (DSM-4 met and GDS > 16)	Posterior lobe of the left cerebellar hemisphere	PFC not covered in study design
Padmanabhan et al., 2019	Lesion location	Varied by five datasets with lesions of different etiologies including stroke; ranges from 28 days to 39 years	Varied by datasets: Neuro-QOL, GDSS, PHQ-9, HADS plus MINI, BDI-II	Binary PSD diagnosis (threshold varied by datasets: Neuro-QOL ≥ 59.9, GDSS ≥ 11, PHQ-9 ≥ 10, HADS ≥ 11 with DSM-4 net, BDI-II ≥ 20)	Negative	No
**Multivariate LSM**						
Grajny et al., 2016	Lesion location	At least 6 months	SADQ	Continuous sum score	Left DLPFC	Yes
Weaver et al., 2021b	Lesion location	Within 1 year, ranges from 1 to 361 days	GDS	Continuous sum score	Right amygdala and pallidum	No
**VBM**						
Shi et al., 2017	GMV	Ranges from 3 months to 1 year	DSM-4 and HDRS	Binary diagnosis (DSM-4 met and HDRS > 17)	Bilateral PFC, limbic system and motor cortex	Yes
Hong et al., 2020	GMV	At least 6 months	DSM-4 and HDRS	Binary diagnosis (DSM-4 met and HDRS > 7)	Left middle frontal gyrus	Yes
**Regional functional activity**						
Egorova et al., 2017	fALFF	3 months	PHQ-9	Both binary diagnosis (PHQ-9 ≥ 5) and continuous sum score	Left DLPFC and right precentral gyrus; left insula	Yes
Goodin et al., 2019	fALFF	3 months	MÅDRS	Binary (MÅDRS > 8)	Frontostriatal, temporal, thalamic, and cerebellar regions	Yes
**Direct connectome analysis**						
Lassalle-Lagadec et al., 2012	rsFC	3 months	HDRS	Continuous sum score	Left middle temporal cortex and precuneus	No
Zhang P. et al., 2014	rsFC	Less than 2 weeks	DSM-4 and HDRS	Both binary diagnosis (HDRS > 7) and continuous sum score	Increased rsFC between the left orbital part of the inferior frontal gyrus and ACC	Yes
Shi et al., 2017	rsFC	Ranges from 3 months to 1 year	DSM-4 and HDRS	Binary PSD diagnosis (DSM-4 met and HDRS > 17)	Decreased rsFC of ACC with PFC, cingulate cortex, and motor cortex, but increased rsFC with the hippocampus, parahippocampal gyrus, insula, and amygdala	Yes
Vicentini et al., 2017	rsFC	Mean: 25 days	DSM-4 and BDI	Continuous BDI score	Left inferior parietal gyrus	No
Balaev et al., 2018	rsFC	7 ± 4 months	ADRS	Continuous sum score	Anterior DMN, salience network, left frontoparietal network	Yes
Egorova et al., 2018	rsFC	3 months	PHQ-9	Both binary diagnosis (PHQ-9 ≥ 5) and continuous sum score	Decreased rsFC between left DLPFC and right supramarginal gyrus	Yes
Sun et al., 2018	rsFC	Mean: 3.6 months	HDRS	Ordinal (3 levels of severity: ≤5, 6–20, and >20)	Decreased rsFC between the parietal-occipital and the frontal areas	Yes
Zhang et al., 2018	rsFC	Mean: 10 days	DSM-4 and HDRS	Both binary diagnosis (HDRS > 7 with DSM-4 met) and continuous sum score	DMN, CCN, AN; left inferior parietal gyrus, the left orbital part of inferior frontal gyrus, and left angular gyrus	Yes
Zhang et al., 2019	rsFC	Less than 6 months	DSM-4 and HDRS	Both binary diagnosis (HDRS ≥ 17 with DSM-4 met) and continuous sum score	Altered rsFC of amygdala with the fronto-limbic-striatal circuit	Yes
Shi et al., 2019	Functional circuit	Ranges from 3 months to 1 year	DSM-4 and HDRS	Binary diagnosis (HDRS ≥ 17 with DSM-4 met)	Ventromedial PFC-ACC-amygdala-thalamus circuit	Yes
Liang et al., 2020	rsFC	3 months	GDS	Binary diagnosis (GDS ≥ 7)	DMN (inferior parietal lobule and dorsal prefrontal cortex)	Yes
Yang et al., 2015	FA; Structural network topology	Within 1 month	DSM-4 and HDRS	Ordinal: major, mild and non-PSD (HDRS ≥ 20, 10–19, <10, respectively)	Wide (including frontal) areas of white matter; a depression-related subnetwork composed of 17 brain regions (including frontal cortex)	Yes
Shen et al., 2019	FA, mean kurtosis	2 weeks	DSM-5 and HDRS	Binary PSD diagnosis based on DSM-5	Bilateral frontal and temporal lobes, genu of corpus callosum	Yes
Xu et al., 2019	Structural network topology	Within 2 weeks	DSM-4 and HDRS	Binary PSD diagnosis based on DSM-4 and HDRS ≥ 7	Disrupted global and local network topologies (involving ipsilesional superior frontal gyrus and middle frontal gyrus, etc.)	Yes
Oestreich et al., 2020	FA, structural network topology	27–82 days	GDS	Binary diagnosis (GDS ≥ 10)	Reward system (including PFC)	Yes
**Indirect connectome analysis**						
Padmanabhan et al., 2019	FDC	Varied by five datasets with lesions of different etiologies including stroke; ranges from 28 days to 39 years	Varied by datasets: Neuro-QOL, GDSS, PHQ-9, HADS plus MINI, BDI-II	Binary PSD diagnosis (threshold varied by datasets: Neuro-QOL ≥ 59.9, GDSS ≥ 11, PHQ-9 ≥ 10, HADS ≥ 11 with DSM-4 met, BDI-II ≥ 20)	A depression circuit centered on left DLPFC and spanning multiple regions (bilateral prefrontal, temporal and parietal)	Yes
Weaver et al., 2021b	SDC	Within 1 year, ranges from 1 to 361 days	GDS	Continuous sum score	Right parahippocampal white matter, right thalamus and pallidum, and right anterior thalamic radiation	Yes (anterior thalamic radiation originates from PFC)

ACC, anterior cingulate cortex; ADRS, aphasic depression rating scale; AN, affective network; BDI, Beck Depression Inventory; CCN, cognitive control network; DLPFC, dorsolateral prefrontal cortex; DMN, default mode network; DSM, Diagnostic Statistical Manual of Mental Disorders; fALFF, fractional amplitude of low frequency fluctuations; FA, fractional anisotropy; FDC, functional disconnection; GDS, Geriatric Depression Scale; GDSS, Geriatric Depression Score Short Form; GMV, gray matter volume; HADS, Hospital Anxiety and Depression Scale; HDRS, Hamilton Depression Rating Scale; LSM, lesion-symptom mapping; MÅDRS, Montgomery-Åsberg Depression Rating Scale; MINI, Mini-International Neuropsychiatric Interview; Neuro-QOL, Neuro-QOL Depression Scale; NRS, Neurobehavioral Rating Scale; PFC, prefrontal cortex; PHQ-9, Patient Health Questionnaire; PSD, poststroke depression; ROI, region of interest; rsFC, resting-state functional connectivity; SADQ; Stroke Aphasic Depression Questionnaire; SDC, structural disconnection; VBM, voxel-based morphometry; VLSM, voxel-based lesion-symptom mapping. *The 17 traditional ROI studies reviewed by Nickel and Thomalla (2017) are not expanded in our table.

More recently, researchers on mental disorders tend to define depressive disorders as a complex symptom network. Both DSM-5 criteria and the sum score of a depression rating scale are based on the traditional “common cause” theory, assuming that depression as an entity causes various symptoms and these symptoms are interchangeable and diagnostically equivalent so that the number or severity of symptoms can be simply added up (Borsboom, 2008; Borsboom and Cramer, 2013; Fried and Cramer, 2017). The depressive symptoms, however, actually interact with each other in complex ways, which has long been common knowledge among clinicians (Borsboom, 2008; Borsboom and Cramer, 2013; Fried and Cramer, 2017). In the psychopathological network theory, mental disorders are conceptualized as dynamic and complex networks of symptoms influencing each other by creating causal pathways and feedback loops (e.g., depressed mood - > insomnia - > fatigue - > depressed mood, in depressive disorders) (Borsboom, 2008; Borsboom and Cramer, 2013; Fried and Cramer, 2017). Depressive symptom networks have been well established in neurologically healthy populations (van Borkulo et al., 2015; Belvederi Murri et al., 2020), and interactions among depressive symptoms may also exist in the stroke population (Ashaie et al., 2021). One recent study modeled poststroke depressive symptoms at three timepoints (discharge and 3 and 12 months after discharge) as networks in which depressed mood was consistently identified as a central symptom and might be responsible for triggering or sustaining the rest of symptoms *via* symptom–symptom interactions (Ashaie et al., 2021). Interestingly, the network structure and connectivity of poststroke depressive symptoms might vary across time after stroke (Ashaie et al., 2021). Accumulating evidence suggests that certain biological processes (e.g., inflammation and focal brain damage) may not be equally related to all depressive symptoms (Moriarity et al., 2022). Investigating risk factors and biomarkers for individual depressive symptoms has become a new research paradigm (Fried et al., 2014, 2020; Jokela et al., 2016; White et al., 2017; Triolo et al., 2021; Moriarity et al., 2022).

## Lesion Location and Poststroke Depression

### Traditional Lesion Analysis

The majority of the evidence about the association between lesion location and PSD is derived from traditional region-of-interest (ROI) analysis in which the lesion is assigned to overlap with a region or not by reviewing the scan without manual segmentation, followed by comparison for the prevalence of PSD in populations defined by the presence or absence of involvement of the ROI. The results from traditional studies before 2017 have been reviewed elsewhere and will not be redundantly described here (Nickel and Thomalla, 2017). In brief, the earliest notion that depression was more likely to be associated with left than with right hemispheric strokes and with lesions in the left anterior brain than with other regions (Robinson and Price, 1982; Starkstein et al., 1987) was not supported by other studies: some concluded with the association between PSD and right hemispheric strokes (MacHale et al., 1998; Wei et al., 2015), while others ended up with no association at all between lesion location and PSD (Carson et al., 2000; Kutlubaev and Hackett, 2014). No conclusion could be drawn regarding the role of lesion location (laterality, anterior–posterior gradient, within specific brain regions, etc.) in the etiology of PSD based on traditional evidence (Nickel and Thomalla, 2017). The traditional ROI approach has its intrinsic limitations. First, the association between the ROI and PSD might be just a coincidence due to the coexisting involvement of other brain regions with common blood supply (so-called consistent error) (Ay et al., 2006; Mah et al., 2014). Second, the traditional approach dichotomizes the study sample with respect to involvement of a relatively large ROI, which precludes point localizations and may result in false negative results due to improper sampling or counterbalancing of effects among multiple small regions within a large ROI (Ay et al., 2006). Another traditional approach called lesion subtraction analysis is also used for lesion-symptom inference (Rorden and Karnath, 2004). Based on the hypothesis that the causative neural substrate is more frequently involved in patients with a specific symptom than in those without, the voxel-wise lesion incidence map of the non-PSD group is subtracted from that of the PSD group showing brain regions more frequently involved in PSD. One lesion subtraction study identified left cerebellar stroke to be correlated with PSD, which was consistent with the result of a subsequent VLSM analysis (Kim et al., 2017). However, the lesion subtraction plot alone may only provide a descriptive result of the possible neural substrate due to lack of statistical tests.

It is not surprising to get inconsistent results from traditional studies (and some other methods in the following text) given the heterogeneity in depression rating scales (Table 1), diverse timepoints of depression assessment since stroke onset (Table 1), small sample size, selection bias (e.g., exclusion for aphasia), different definitions of lesion location, inadequate imaging quality, combining lesions of various etiologies into a single analysis, and so on (Robinson and Jorge, 2016; Nickel and Thomalla, 2017; Towfighi et al., 2017). Of note, the interval between stroke onset and depression assessment might be an important factor exerting influence on the results of the traditional approach (and other methods as well). A longitudinal study focusing on depression at specific stages of stroke found that the association of PSD with left anterior lesions appeared to be a transient phenomenon restricted to the acute stage and the laterality tended to reverse to the right hemisphere in the long-term follow-up (Astrom et al., 1993). Another independent study also suggested that only in-hospital depression was associated with lesions in the left anterior hemisphere and depression at 1–2 years poststroke was associated with right-hemisphere lesion volume and lesion proximity to the occipital pole (Shimoda and Robinson, 1999). A meta-analysis with stratification by time of assessment showed that the association between right hemispheric stroke and PSD only existed at the subacute stage of stroke (Wei et al., 2015). It is conceivable that the neuroanatomic substrates of PSD might change over time or there might be distinct mechanisms for PSD at different stages of stroke (Astrom et al., 1993; Shimoda and Robinson, 1999). Therefore, studies mixing patients of various stages poststroke tend to produce inconsistent and unreliable results and performing PSD assessments at homogeneous stages after stroke might be helpful in future studies. Although inconclusive, some researchers still believe that there is an association between left frontal strokes and PSD within the first 2 months after stroke based on some converging evidence (Astrom et al., 1993; Shimoda and Robinson, 1999; Rajashekaran et al., 2013; Robinson and Jorge, 2016). The role of the left prefrontal cortex (PFC) in PSD was further supported by multiple clinical trials showing that repetitive transcranial magnetic stimulation (rTMS) targeted at the left dorsolateral PFC (DLPFC) can significantly alleviate the symptoms of PSD and vascular depression (Jorge et al., 2004; McIntyre et al., 2016; Gu and Chang, 2017; Shen et al., 2017; Frey et al., 2020).

### Voxel-Based and Multivariate Lesion-Symptom Mapping

The general procedure of VLSM analysis involves lesion segmentation on computed tomography (CT) or magnetic resonance imaging (MRI) scans, spatial normalization of lesion masks, mass-univariate statistical tests voxel by voxel for the association between lesion status at a given voxel and presence or severity of the symptom of interest, correction for multiple comparisons to control false positive rates, and interpretation of the significant clusters. Three lesion-symptom mapping (LSM) studies on PSD before 2017 did not provide consistent results, as described in the previous review (Nickel and Thomalla, 2017). The first study showed negative results, and another two identified lesions in the left cerebellum and left DLPFC, respectively, as the neural substrates of PSD (Gozzi et al., 2014; Grajny et al., 2016; Kim et al., 2017). These are considered as pilot exploratory LSM studies with small sample size (*n* < 100) and therefore low statistical power (Nickel and Thomalla, 2017). Another study in 2019 which performed VLSM in five datasets (total *n* = 461) with focal brain damage of various etiologies (including stroke) failed to find any lesion location to be correlated with depression after brain damage (but did identify a functional depression circuit *via* connectome analysis) (Padmanabhan et al., 2019). Generally, only voxels with lesion incidence above a certain threshold (e.g., at least 10 patients or 5% of patients, indicating sufficient lesion affection) could be included in VLSM to ensure reasonable statistical power and anatomical validity (Medina et al., 2010; Sperber and Karnath, 2017). Small sample size tends to provide insufficient lesion coverage, leaving those infrequently involved brain regions with lower statistical power or being unexplored (Kimberg et al., 2007; Gozzi et al., 2014). Indeed, a large sample size will be required if we want to perform whole-brain VLSM analysis without predefined ROIs, considering the recent evidence that about 3,000 subjects were needed to achieve a lesion coverage of 86% of total brain voxels (Weaver et al., 2021a). The original VLSM approach with mass-univariate tests still suffers the aforementioned consistent error originating from collateral vasculature (Mah et al., 2014; Karnath et al., 2018). The latest machine learning-based multivariate LSM approaches, believed to be able to overcome this limitation, seem as promising alternatives in future studies (Mah et al., 2014; Karnath et al., 2018). One multivariate LSM study mentioned above applied support vector regression-based LSM (SVR-LSM) and found the association between lesions in the left DLPFC and severity of poststroke depressive symptoms (PSDS; Zhang Y. et al., 2014; Grajny et al., 2016). Another recent SVR-LSM study found that infarcts in the right amygdala, right pallidum, and right hippocampus were associated with PSDS (Weaver et al., 2021b). External validation confirmed the association between infarcts in the right amygdala and pallidum, but not the right hippocampus, and PSDS (Weaver et al., 2021b). Another latest method named multivariate sparse canonical correlations technique (SCCAN), generally superior to the univariate approach at small sample sizes, may also be recommended in future studies (Pustina et al., 2018). Some neural substrates unlikely to be identified with the univariate approach may be unveiled with multivariate LSM which integrates lesion information from multiple voxels simultaneously in lesion-symptom inference (Karnath et al., 2018). Multivariate approaches are preferred if we have good reasons to assume that the symptom of interest is represented in an extensively distributed brain network, as in these cases the ability of the univariate approach to detect all of the network modules may be limited (Karnath et al., 2018; Xu et al., 2018). Based on current LSM studies showing non-convergent results, the role of lesion location in the etiology of PSD still warrants further investigation with large datasets and advanced methodologies.

## Regional Structural and Functional Abnormalities and Poststroke Depression

Gray matter atrophy may serve as a transdiagnostic neural substrate for various mental illnesses (Goodkind et al., 2015). Voxel-based morphometry (VBM) is able to evaluate the intergroup difference of gray matter volume (GMV) at the voxel level using high-resolution structural MRI (sMRI; Ashburner and Friston, 2000). The procedure of VBM involves spatially normalizing and segmenting sMRI images into the same standard space (Ashburner and Friston, 2000). The gray matter segments are smoothed so that each voxel represents the average of itself and its neighbors (Ashburner and Friston, 2000). Parametric statistical tests are performed at the voxel level, followed by corrections for multiple comparisons with the theory of random fields (Ashburner and Friston, 2000). One study performed VBM analysis in 30 first-ever ischemic frontal stroke patients showing that decreased GMV in PSD was mainly observed in the prefrontal-limbic system and motor cortex compared with non-PSD patients (Shi et al., 2017). The involved limbic structures were mainly located in the right hemisphere, and the PFC showed a decreased GMV in both hemispheres (Shi et al., 2017). Recent evidence from another VBM study in 23 PSD and 33 non-PSD subcortical stroke patients suggested that the PSD group had significantly decreased GMV in the left middle frontal gyrus (MFG; Hong et al., 2020). Together with the clinical-demographic variables, the MFG’s GMV prediction model was able to distinguish PSD from non-PSD with high sensitivity and specificity (Hong et al., 2020). The results from VBM studies are in accordance with the aforementioned role of the frontal cortex in PSD, as well as the frontal-limbic model which is well-recognized in depressive disorders such as major depressive disorder (MDD) and vascular depression (Taylor et al., 2013; Lai, 2021).

Resting-state functional MRI (rs-fMRI), reflecting brain activation *via* blood oxygenation level-dependent (BOLD) signal, has been widely used in research of neuropsychiatric disorders including stroke and depression (Ovadia-Caro et al., 2014; Oathes et al., 2015). Several measurements, such as amplitude of low-frequency fluctuations (ALFF) and fractional ALFF (fALFF), were developed to reflect the characteristics of spontaneous brain activity within a brain region (Zang et al., 2007; Zou et al., 2008). One rs-fMRI study performed in 64 participants at 3 months poststroke found significantly higher fALFF in PSD in the left DLPFC and the right precentral gyrus compared with non-PSD patients and a significant association between higher PSD severity and higher fALFF in the left insula (Egorova et al., 2017). The aberrant regional brain activity could be a more sensitive feature than lesion location and volume to characterize PSD in small samples (Egorova et al., 2017). Another rs-fMRI study identified a significant correlation between PSD severity and fALFF in frontostriatal, temporal, thalamic, and cerebellar regions (Goodin et al., 2019). These fMRI results also provided evidence for the role of the frontal-limbic system in PSD. However, functional neuroimaging is time-consuming and vulnerable to head motions which is difficult to control in stroke patients (Ovadia-Caro et al., 2014; Fox, 2018). Another major limitation of fMRI studies is that they can hardly be used for causal inference (Fox, 2018). Unlike visual or voxel-based lesion analyses which have a clear temporal order between lesion and PSD, fMRI analysis typically has neuroimaging performed at the time of or after PSD diagnosis for a case–control comparison. Therefore, whether this functional difference between PSD and non-PSD is causative, or whether it is a reactive or adaptive alteration to depression, remains elusive (Fox, 2018). The results from fMRI analyses should be regarded as correlation rather than causation (Fox, 2018). Future studies with longitudinal design might unravel the potential causal relationship between gray matter atrophy, regional functional abnormality, and development of PSD.

## Brain Network Disruption and Poststroke Depression

Based on large amounts of lesion studies, the lesions of PSD patients fail to overlap in a single brain region. It has long been discovered that some neuropsychiatric symptoms can result from dysfunctions of anatomically intact brain regions which are distant but connected to the lesion (“diaschisis” phenomenon) (Carrera and Tononi, 2014). In the era of brain networks and human connectome, we have good reasons to assume that PSD might be a complex disconnection syndrome resulting from disruption of networks of interacting brain regions (Gong and He, 2015). There are two types of brain connectivity being explored. Functional connectivity (FC) is measured by the correlation of the fMRI BOLD time series between brain regions, and structural (or anatomical) connectivity (SC) can be derived from diffusion tensor imaging (DTI) in which water diffuses more freely along white matter fibers than across them (Fox and Raichle, 2007; Jbabdi et al., 2015). Brain networks can be analyzed at different levels: microscale (single neuron and synapses), mesoscale (neuronal groups), and macroscale (brain regions and inter-region pathways) (Sporns et al., 2005). Quantitative analysis, especially graph theory analysis, can reveal important features of complex networks such as highly connected hubs, modularity, and small-world topology (Bullmore and Sporns, 2009; He and Evans, 2010). Importantly, these quantifiable network features were found to change in normal development, aging, and various neuropsychiatric disorders (He and Evans, 2010; Menon, 2011; Ovadia-Caro et al., 2014). With both FC and SC techniques, complex symptoms like PSD that transcend localization to single brain regions may be mapped to widely distributed brain networks.

### Direct Connectome Analysis

Typically, the FC is directly derived from each patient’s fMRI scans. One study in frontal stroke patients performed connectivity analysis with the anterior cingulate cortex (ACC) as the seed found decreased resting-state FC (rsFC) with the PFC, cingulate cortex, and motor cortex, but increased rsFC with the hippocampus, parahippocampal gyrus, insula, and amygdala, in the PSD group (Shi et al., 2017). Another study in temporal stroke patients found that the left amygdala had increased rsFC with the bilateral precuneus and right orbital frontal lobe but decreased rsFC with the right putamen in PSD compared with non-PSD patients (Zhang et al., 2019). The right amygdala had increased rsFC with the right temporal pole, right rectus gyrus, and left orbital frontal lobe but decreased rsFC with the right primary sensory area (S1) (Zhang et al., 2019). The amygdala’s rsFCs with the right orbital frontal cortex, right insula, and right cingulate cortex were correlated with the HDRS score (Zhang et al., 2019). A multivariate Granger causality analysis in right frontal ischemic stroke patients found an emotional circuit (composed of ventromedial PFC, ACC, amygdala, and thalamus) to explain the network alterations in PSD (Shi et al., 2019). The DLPFC could predict the activity of the ACC *via* the temporal pole, and the activity of the insula could be regulated negatively by the thalamus *via* ACC (Shi et al., 2019). Altered rsFC of the default mode network (DMN), cognitive control network (CCN), and affective network (AN) was observed in PSD compared with non-PSD patients and normal comparisons (NC; Zhang et al., 2018). The left orbital part of the inferior frontal gyrus, the left inferior parietal gyrus, and the left angular gyrus (which indicated altered rsFCs) were significantly correlated with HDRS scores in PSD patients (Zhang et al., 2018). Dysfunction of the AN in PSD was also observed in another study which found that the rsFCs of the left inferior temporal gyrus, the left orbital part of the inferior frontal gyrus, and the right triangular part of the inferior frontal gyrus were increased with the ACC in PSD compared with non-PSD stroke patients (Zhang P. et al., 2014). Moreover, the rsFC between the left orbital part of the inferior frontal gyrus and ACC was positively correlated with PSD severity (Zhang P. et al., 2014). As for the CCN, PSD was associated with decreased rsFC between left DLPFC and right supramarginal gyrus (Egorova et al., 2018). Graph theory analysis found that the DMN configuration (especially at core hubs such as dorsal PFC and inferior parietal lobule) might be more essential in the pathogenesis of PSD than stroke lesions (Liang et al., 2020). RsFC between anterior DMN and salience network positively correlated with PSD severity, and rsFC between anterior DMN and left frontoparietal network decreased after treatment of PSD (Balaev et al., 2018). Earlier evidence suggested that DMN dysfunction soon after stroke was predictive of PSD severity at 3 months poststroke (Lassalle-Lagadec et al., 2012). Both PSD and poststroke anxiety was found to be associated with DMN disruption (Vicentini et al., 2017). Depression symptoms were found to be correlated with increased rsFC in the left inferior parietal gyrus (Vicentini et al., 2017). Other than fMRI, electroencephalography (EEG) can also be used for rsFC analysis. In one study using mutual information-based graph theory analysis on EEG data, stroke patients showed a decreasing trend in the rsFC between the parietal–occipital and the frontal areas as PSD severity increased (Sun et al., 2018). These functional studies provided promising evidence for the role of emotion- and cognition-related networks in PSD, of which the clinical applicability is yet to be determined considering the complexity of brain networks. Furthermore, most rs-fMRI studies lack the ability of causal inference but reveal a correlation instead as discussed in the previous section (Rorden and Karnath, 2004; Fox, 2018). A functional alteration may be the result of compensation for PSD rather than its cause, and treatment strategies suppressing the alteration could make the symptom worse (Fox, 2018). Targeting the region where brain activity is correlated with but not causally related to PSD may have no effect at all (Fox, 2018). These ambiguities, along with the susceptibility to motion artifacts, make it difficult to translate fMRI correlates directly with therapeutic targets (Rorden and Karnath, 2004; Ovadia-Caro et al., 2014; Fox, 2018).

Direct SC analysis is typically based on each patient’s DTI scans. SC at the neuronal level is the densely distributed axonal streamlines within white matter to connect gray matter regions. Regions that are structurally connected tend to also be functionally connected; however, FC presents a different pattern than SC due to the impact of extensive polysynaptic connections (Fox and Raichle, 2007; Fornito et al., 2015). Recent evidence suggested that the functional brain network dysfunction after stroke can be explained by structural disconnection (SDC; Griffis et al., 2019, 2020). Therefore, SDC could be a more upstream neural substrate of PSD than functional network dysfunction. One study used fractional anisotropy (FA) to reflect white matter integrity and found that the mean FA of the intact areas of stroke-lesioned tracts was lower than that of completely intact fiber tracts (Yang et al., 2015). FA reductions were observed in wide areas of white matter in PSD compared with non-PSD (Yang et al., 2015). Graph theory analysis revealed that decreased local efficiency of a depression-related subnetwork was a significant risk factor for major depression poststroke (Yang et al., 2015). Another study suggested that aberrant global and local structural network topologies might contribute to the development and severity of PSD (Xu et al., 2019). Recent evidence showed that reduced FA, along with increased extracellular free water (a marker of neuroinflammation) and GMV loss, in the reward system was predictive of PSD (Oestreich et al., 2020). However, one major limitation of FA and tractography analysis lies in their susceptibility to complex brain architecture such as fiber crossing (Mori and van Zijl, 2002; Jeurissen et al., 2013). For example, FA may be actually increased after microstructural disruption in regions of fiber crossing. The diffusion tensor model can account only for the Gaussian diffusion process which is not always the case in the human brain and the fiber crossing issue should not be overlooked in future DTI studies (Jeurissen et al., 2013). Diffusion kurtosis imaging (DKI), applying a more complex model than DTI to quantify non-Gaussian water diffusion and reveal complex microstructures, was also used to investigate the role of white matter integrity in PSD (Jensen et al., 2005). DKI may be useful for unveiling abnormalities in isotropic structures, such as fiber crossing and gray matter, where DTI is less applicable (Jensen et al., 2005). One DKI study found that the FA value of the left frontal lobe and the mean kurtosis (MK) value of the bilateral frontal and temporal lobes and genu of the corpus callosum were significantly decreased in PSD compared with non-PSD patients and NC (Shen et al., 2019). Concerning the relationship between the structural and functional networks in PSD, one study identified the SC-FC coupling as a potential biomarker of PSD (Zhang et al., 2021).

### Indirect Atlas-Based Connectome Analysis

Thanks to great efforts such as the Human Connectome Project, connectome atlases of averaged FC and SC patterns from hundreds or thousands of normal populations are now readily available (Glasser et al., 2016). Beyond lesion analysis, we can now combine lesion data with connectome atlases to map the network substrates for PSD without specialized neuroimaging for each individual (Boes et al., 2015; Fox, 2018).

Lesion-network mapping (LNM), a technique to combine lesion with normative functional connectome, generally involves three steps: translating the three-dimensional lesion into the standard space; estimating FC of the lesion with the rest of the brain using normative functional connectome atlas; and overlapping or statistically comparing FC maps derived from patients with the same symptom to identify regions common to the syndrome of interest (Boes et al., 2015; Fox, 2018). LNM is becoming increasingly popular in recent years to map the strategic network substrates of various conditions such as PSD, poststroke behavioral deficits, and mania after brain damage (Padmanabhan et al., 2019; Cotovio et al., 2020; Salvalaggio et al., 2020). A recent LNM study suggested that the lesion locations associated with depression after focal brain damage could be mapped to a human depression circuit centered on left DLPFC (Padmanabhan et al., 2019). Three rTMS targets reported to be effective in treating PSD fell within this circuit (Padmanabhan et al., 2019). A similar method named “dys-connectome” or disconnectome has been used to map the structural network substrates for PSD, poststroke behavioral deficits, poststroke fatigue, and so on (Salvalaggio et al., 2020; Ulrichsen et al., 2021; Weaver et al., 2021b). Powerful tools, such as BCBtoolkit and Lesion Quantification Toolkit, allow researchers to indirectly estimate SDCs with the lesions embedded in the structural connectome atlas and then perform disconnection-symptom inference at tract or voxel levels (Foulon et al., 2018; Griffis et al., 2021). Recent evidence from a multivariate disconnection-symptom mapping study found that SDCs in the right parahippocampal white matter, right thalamus and pallidum, and right anterior thalamic radiation were significantly associated with PSDS (Weaver et al., 2021b).

The atlas-based approach, without need for specialized neuroimaging, may prove a broadly applicable and versatile way for understanding the neural basis of neuropsychiatric symptoms after focal brain damage (Boes et al., 2015; Fox, 2018). However, the atlas-based approach does not necessarily reveal the actual disconnection patterns (Weaver et al., 2021b). The connectome architecture has its intrinsic individual variability (Mueller et al., 2013). It should also be noted that concomitant conditions, other than the stroke lesion, may have impact on brain networks. For example, cerebral small vessel disease (CSVD) is common in elderly stroke patients and can increase the risk of PSD (Tang et al., 2010, 2014; Bae et al., 2019; Liang et al., 2019). Moreover, structural network disruption was observed in CSVD compared with NC (Xie et al., 2017; Lawrence et al., 2018). The impact on the brain network from concomitant factors with inter-person variability may only be taken into account by direct connectome analysis. In terms of causal inference, lesion network maps span several regions at all levels of the neuro-axis and the extent to which any of them are causal to the symptoms is unknown and would require other complementary approaches. The indirect nature of the atlas-based approach requires confirmation and complementation by other methods including direct mapping of residual structural and functional connections in the patients (Salvalaggio et al., 2020; Bobes et al., 2021; Boes, 2021).

## Discussion: Future Directions and Clinical Implications

Studies reviewed in this work are summarized in Table 1 regarding study design and findings. From the time of lesion analysis to the era of brain networks, our knowledge and understanding of the neural substrates for PSD are increasing. Pooled evidence (Table 1) from traditional lesion analysis, univariate or multivariate LSM, regional structural and functional analyses, and connectome analysis, to some extent, echoes the frontal-limbic theory described in multiple depressive disorders including MDD and vascular depression (Taylor et al., 2013; Robinson and Jorge, 2016; Lai, 2021). Despite differences in etiology and symptom profile (da Rocha e Silva et al., 2013; Albert, 2018), PSD and MDD may share the common neural basis. As shown in Table 1, the PFC as replicated across studies of various methodologies may be the structure most heavily implicated in the pathogenesis of PSD. The role of PFC in depressive disorders has been confirmed by multiple clinical trials, showing that rTMS targeted at the left DLPFC can significantly alleviate the symptoms of PSD, vascular depression, and MDD (Jorge et al., 2004; O’Reardon et al., 2007; George et al., 2010; McIntyre et al., 2016; Gu and Chang, 2017; Shen et al., 2017; Frey et al., 2020; Sackeim et al., 2020). We believe that the PFC is most promising as the avenue for future research or the target for treatment of PSD and other depressive disorders. The distinct roles of subregions of the PFC in mood regulation (Koenigs et al., 2008) and the optimal stimulation target within PFC for neuromodulation therapy are yet to be scrutinized. To date, the definite neural substrates of PSD along with their clinical applications still entail further investigation. Patients with focal brain damage have long been proper study samples for understanding human brain-behavior mechanisms (Fox, 2018; Vaidya et al., 2019), and the findings from research on PSD may also be generalized to depressive disorders in neurologically healthy population.

To improve inter-study consistency and accumulate further evidence, we provide some suggestions for future studies in the field. First, in addition to DSM-5 criteria, researchers may apply a validated depression rating scale with a consistent cutoff value for PSD diagnosis (Berg et al., 2009). Scales that can be generalized to patients with aphasia may be recommended to reduce bias in patient selection (Cobley et al., 2012). Second, serial assessments of depression status (and neuroimaging if necessary) at acute, subacute, and chronic stages of stroke are performed to uncover the potentially dynamic neural substrates of PSD across various stages after stroke. Third, LSM and connectome studies in multicentric large datasets with cross or external validation are recommended to derive comprehensive maps for the strategic lesion locations and disconnection patterns for PSD. Fourth, the neural substrates of PSD at the individual symptom level are uncovered, rather than simply using a binary PSD diagnosis or a continuous sum score. Note that depressive symptoms may have different risk factors and biomarkers (Fried et al., 2014; Moriarity et al., 2022), and the neural substrates of PSD may also be symptom-specific. The symptom-specific anatomical correlates, as well as the complex and dynamic pattern of symptom-symptom interactions, may help us better understand the brain-behavior mechanisms of PSD. Fifth, preregistration of anatomical hypotheses may add rigor to this field. The anatomical hypothesis and analysis plan before observing the research outcomes are defined to prevent problematic research practices such as false positive results from p-hacking and publication bias (Nosek et al., 2018, 2019).

Understanding the neural substrates for PSD is of great predictive value. For example, the strategic lesion locations and disconnection patterns for PSD can be used to develop prediction models for risk stratification and early intervention (Munsch et al., 2016; Salvalaggio et al., 2020; Weaver et al., 2021a,b). Only a few PSD prediction models based on routine clinical-demographic data have been developed and validated (de Man-van Ginkel et al., 2013; Hirt et al., 2020). Stroke severity, physical disability, history of depression, and cognitive impairment have been the most well-recognized predictors of PSD (Towfighi et al., 2017). Sex, age, education level, social support, and personality traits (e.g., neuroticism) are also considered potential (although less clear) predictors of PSD (Towfighi et al., 2017; Chun et al., 2021). A previous study found that both functional dependence and neuroanatomical measures were correlated with more PSD symptoms (Singh et al., 2000). Although inferior frontal lesion location was a risk factor of PSD, the degree of functional dependence imparted the greatest risk (Singh et al., 2000). To date, no imaging predictor is found to outperform classic clinical-demographic variables in PSD prediction. Considering the biopsychosocial multifactorial nature of PSD, incorporating neural substrates into clinical-demographic predictors might be a good choice in PSD prediction (Munsch et al., 2016; Towfighi et al., 2017; Hong et al., 2020). Moreover, advances in methodology as detailed above may provide new promising imaging predictors for PSD in the future. The neural substrates of PSD also have significant preventive and therapeutic value. Many studies (Table 1) can provide convergent evidence for the role of PFC in PSD and therefore the effectiveness of rTMS targeted on left DLPFC. Future studies on brain networks might find personalized preventive or therapeutic targets for PSD based on human connectome (e.g., an emerging technique called connectomic neuromodulation) (Cocchi and Zalesky, 2018; Fox, 2018; Horn and Fox, 2020; Siddiqi et al., 2020). The optimal individualized stimulation site may provide enhanced response to neuromodulation therapy for depression (Cash et al., 2019, 2021). Of note, distinguishing adaptive brain alterations to PSD from causative neural substrates is of great importance in future studies because strategies to inhibit the adaptive changes may lead to exacerbation of PSD (Fox, 2018). Moreover, the techniques reviewed here for uncovering neural substrate can also be extended to clinical research for a broad range of disorders with focal brain damage.

## Author Contributions

SZ and ZZ led the study and reviewed and edited the final manuscript. GL, WS, JM, XQ, YL, YW, and HW searched and collected the literature. CP prepared the original draft. All authors contributed to the article and approved the submitted version.

## Conflict of Interest

The authors declare that the research was conducted in the absence of any commercial or financial relationships that could be construed as a potential conflict of interest.

## Publisher’s Note

All claims expressed in this article are solely those of the authors and do not necessarily represent those of their affiliated organizations, or those of the publisher, the editors and the reviewers. Any product that may be evaluated in this article, or claim that may be made by its manufacturer, is not guaranteed or endorsed by the publisher.
